# Anxiety among pregnant women in Addis Ababa, Ethiopia

**DOI:** 10.1371/journal.pone.0318718

**Published:** 2025-02-14

**Authors:** Hanna Melesse, Tigest Shifraw, Yemane Berhane

**Affiliations:** 1 Department of Epidemiology and Biostatistics, Addis Continental Institute of Public Health (ACIPH), Addis Ababa, Ethiopia; 2 Department of Reproductive Health and Population, Addis Continental Institute of Public Health (ACIPH), Addis Ababa, Ethiopia; Arba Minch University, ETHIOPIA

## Abstract

**Background:**

Anxiety is one of the most prevalent mental health problems during pregnancy which result in various maternal and newborn complications affecting the health and well-being of the mother and the baby. In countries like Ethiopia, anxiety among pregnant women was not well explored, limiting the development of informed interventions. Thus, this research aims to assess the magnitude of anxiety and associated factors among pregnant women visiting ANC at public health centers in Addis Ababa, Ethiopia.

**Method:**

We conducted an institutional-based cross-sectional study. Pregnant women who visited antenatal care (ANC) from April 1–14, 2021, were recruited from seven public health centers in Addis Ababa, Ethiopia. Data were collected using a structured questionnaire by trained data collectors. Poisson regression with a robust error variance estimate was used to calculate the prevalence ratio to identify the associated factors; the results were presented with an adjusted prevalence ratio (APR) and a 95% CI.

**Result:**

A total of 323 pregnant women were recruited from the ANC clinics of public health centers in Addis Ababa. The overall prevalence of anxiety disorder was 12.70%, 95% CI (9.00%–16.10%). The prevalence risk of anxiety was higher among women who reported unplanned pregnancy compared to those who had planned pregnancy (APR 1.99, CI 1.04–3.81), and being single was associated with a higher prevalence risk than being married (APR 2.29, CI 1.14–4.59).

**Conclusion:**

More than 1 in 10 women had anxiety during pregnancy. The prevalence of anxiety was about two-fold higher among single mothers and among mothers who had an unplanned pregnancy. The study suggests the importance of integrated mental health assessment and services in regular antenatal care, with an emphasis on identifying and caring for women at a disadvantage due to social and obstetric factors.

## Introduction

Globally, maternal mental health is one of the concerning public health problems. According to the WHO, 10% of pregnant women develop mental health problems [1]. Anxiety is one of the most prevalent mental health problems during pregnancy as a result of the physical and psychological changes [2,3]. However there is a propensity to overlook anxiety during pregnancy in favor of maternal and fetal physical health and to mistakenly link emotional problems to physical and hormonal changes that take place during pregnancy [3].

Anxiety during pregnancy can negatively affect the health of the mother and the well-being of the baby [4]. It is associated with miscarriage [5], preterm delivery, low birth weight, prolonged labor, delay in the initiation of breastfeeding, and increased neonatal mortality [4,6,7]. Additionally, perinatal services are more likely to be accessed late, they are less likely to attend antenatal appointments frequently, and they are less likely to have regular scans. Antenatal anxiety has also been linked to poor nutrition and weight gain, increased alcohol consumption, substance abuse, and smoking [3]. Moreover, prenatal anxiety could be a precursor to postpartum depression, which in turn affects parenting [8].

Worldwide, the prevalence of anxiety varies from 11.4% to 63% [9]. A meta-analysis done on data from 102 studies conducted in 34 countries of the world found the overall estimate of anxiety in pregnant women to be 15.3% [10]. The overall report of anxiety during pregnancy is generally higher in lower- and middle-income countries (LMIC) compared to high-income countries [11].Women in LMIC settings are exposed to a high level of stressful events, increasing their vulnerability to anxiety with every additional stressful experience [11]. The self-reported prevalence of anxiety was 29.2% in a meta-analysis conducted in LMIC [12]. In Ethiopia, studies conducted on anxiety indicate the prevalence ranges from 4.20% [13] to 43.9% [9].

The risk factors for anxiety include obstetric-related events such as unwanted pregnancy, previous pregnancy complications, previous pregnancy loss, and fear of childbirth [11]. Other socio-demographic, economic, and medical factors for antenatal anxiety include low educational level, loss of a husband, unemployment, low family income, heavy household responsibilities, family history of mental illness, substance abuse, chronic medical illnesses, and intimate partner violence [14–16]. It is also linked to social isolation and a lack of social support [17,18]. Some other stressors such as the COVID-19 pandemic can cause unprecedented challenges to mental health [19] due to fear of acquiring infection, the emotional disturbances created by the death of people, the significant changes to daily lives, lack of physical contact with family members, and its negative economic impact [20–22].

This research aims to assess the magnitude of anxiety and associated factors in pregnant women visiting ANC at public health centers in Addis Ababa, Ethiopia. In countries like Ethiopia anxiety among pregnant women was not well explored, limiting the development of informed interventions. Conducting the study in the local context will provide a better understanding of the problem for health care providers and policymakers to enhance and implement evidence-based strategies to identify and support pregnant women experiencing anxiety during ANC visits.

## Methods

### Study setting

Addis Ababa is the capital city of Ethiopia, with a population size of 3,433,999 according to health and health-related indicators of 2017. The city is administratively divided into 11 sub-cities. The public health facilities providing antenatal care in Addis Ababa include 97 health centers and 11 hospitals. In addition, there are over 900 private clinics and 25 private hospitals in the city [23]. Antenatal care is one of the exempt services provided in Ethiopia [24]. According to the 2019 Ethiopian demographic health survey report, 81.8% in Addis Ababa had four or more ANC visits for their most recent live birth.

### Study design

The study was a facility-based, cross-sectional study.

### Source population

The source population were pregnant women receiving antenatal care service in public health centers of Addis Ababa, Ethiopia.

### Study population

The study population consisted of pregnant women visiting the ANC at the selected public health centers in April 2021. Participants age 18 and above, willing to participate and give written consent, were included. The exclusion criteria included pregnant women with emergency medical conditions and emergency obstetric complications.

### Sample size

A single population formula was used to calculate the sample size necessary to determine the magnitude of anxiety during pregnancy with the following assumptions: a prevalence of 32.2% [25], a 95% confidence level, a margin of error of 0.04, Z = 1.96, and a 5% non-response rate. The calculated sample size was corrected for the finite population size, and the required sample size for this study was 319.

### Sampling procedures

We randomly selected two sub-cities from Addis Ababa, Arada, and Gulele, for this study. Then, we randomly selected 7 health centers from the two sub-cities after obtaining the complete list from the city administration health office. The sample size calculated for the study was proportionally allocated to the seven health centers based on their ANC client load. Finally, all pregnant women visiting the ANC during the data collection period were consecutively recruited for the study.

### Study variables and measurements

Data were collected using a questionnaire prepared in English and translated into Amharic (the country's official language). The questionnaire included socio-demographic questions, general obstetric questions, the Hospital Anxiety subscale (HADS-A), and the Multidimensional Scale of Perceived Social Support (MSPSS). The data was collected by using electronic devices (tablets) with ODK (open data kit) software, which allows creating a questionnaire that can be used on a mobile phone or tablet running the Android operating system. It doesn't need an internet connection for collecting the data but needs a connection for sending the aggregated data to the dedicated server.

The Hospital Anxiety and Depression Scale was developed as a self-assessment tool to identify anxiety and depression in non-psychiatric hospital departments in patients aged 16–65 years [26]. It was designed for use in hospitals, but many studies have shown that it can also be used in primary care settings, communities, antenatal clinics, and well-person screening [27]. The scale is simple to use, takes 2–5 minutes to complete, and can be used by non-psychiatric nurses and physicians. The Amharic version of the scale showed acceptable reliability and validity [28]. In this study, the HADS-A had good reliability with Cronbach’s alpha score of 0.76. The scale has seven items for anxiety, each item has a minimum score of 0 and a maximum score of 3, which gives a maximum total score of 21 for anxiety disorder. A total score ≥8 was defined as having anxiety [26].

The MSPSS was developed to measure the perceived social support with 12-item ratings with 7-point Likert-type scaling. It measures the adequacy of perceived support from family, friends, and significant others. The scale had excellent reliability with Cronbach’s alpha score of 0.90. The average score was taken for categorizing the group: 1 was the minimum average score and 7 was the maximum average score. High support (5.1–7), moderate support (3–5), and low support (1–2.9). [29]

### Data collection procedures

Data collection was conducted from April 1–14, 2021, at the selected public health centers in Addis Ababa, Ethiopia. Data collection started after communicating with the responsible body at the Addis Ababa city administration health bureau and health centers. The data was collected by nurses and health officers with previous experience in health data collection. Data collectors received training on the contents of the questionnaire, the ethical conduct of the study, and the use of ODK. The data were collected in a comfortable and private space available at the health centers. Interviews were conducted at the exit from the regular antenatal care services.

### Data quality control

The data was collected using tablets to improve the quality by reducing missing data and errors, reducing the need for processing and cleaning. One day of training on the contents of the questionnaire, the ethical conduct of the study, and a brief discussion on how to use the ODK on the tablet was given by the investigator. The tool was also pretested one week before the main survey to check for clarity of the contents and edited accordingly. The data collection was supervised with a frequent visit to the sites of data collection and observation of the conduct of the interview and completeness of the forms. In addition to this the data sent to the server by each data collector was regularly checked.

### Data management and analysis

Data cleaning, coding, and analysis were done using Stata version 14. Descriptive statistics such as frequency and percentage were used to present the characteristics of participants.

We used Poisson regression with robust error variance (modified Poisson regression) to examine the association between the outcome variable and the independent variable. The commonly used model to assess association when the outcome variable is dichotomous is the logistic regression model which directly gives the adjusted odds ratio [30]. In this model, when the outcome is uncommon, the difference between the odds ratio and relative risk is insignificant. However, when the outcome is common (prevalence > 10%) the logistic regression model overestimates the prevalence ratio [31,32]. In such cases, it is recommended to use the Poisson regression with a robust error variance, which better approximates the prevalence ratio [33–35]. It is inappropriate to model binary outcomes in the traditional Poisson regression model because it violates the assumption for a Poisson distribution. Therefore, to address this lack of Poisson distribution in a binary outcome, a robust variance estimator (sandwich variance estimator) is used. It is unnecessary to make a Poisson distribution assumption for the outcome when using the robust Poisson regression [34]. Moreover, Poisson regression with a robust error variance approach for binary data is simple to use, doesn't need modifying the data, and directly provides the prevalence ratio, which is easily interpretable [36].

To ascertain the magnitude and direction of the association between the independent and the outcome variables, the unadjusted prevalence ratio (PR) with a 95% confidence interval (CI) was calculated. The multi-variable model was designed to account for the possible influence of confounding variables. The variables that showed significant association in the unadjusted analysis and are pertinent and referenced in various literature were included in the adjusted model. A probability value of p ≤ 0.05 was used as the level of significance. The coefficients from the unadjusted and adjusted regression models were presented as PR and APR, respectively, with a 95%CI.

### Ethical consideration

Ethical approval for conducting this study was obtained from the ethical review board of the Addis Continental Institute of Public Health with ethical clearance number Ref. No.: ACIPH-MPH/013/13 and the Addis Ababa City Administration Health Bureau with ethical clearance number Ref. No.: A/A/H/9341/227. Written, informed consent was obtained from all participating pregnant women after clearly describing the purpose of the study. For those who are unable to read, the form was read aloud to them. For those who were unable to sign, fingerprints were taken and witnessed by an impartial, literate witness. They were informed that they were free to withdraw their consent at any point in the research process. Interviews were conducted in private spaces to ensure privacy and confidentiality. Appropriate prevention methods for COVID-19, like wearing masks, maintaining hand hygiene, and keeping distance, were implemented during data collection.

## Result

A total of 325 pregnant women were eligible for this study during the study period, of which 323 (99.38%) agreed to participate in this study. The participants were in the age range of 18–39. The 202 (62.50%) of the participants completed secondary and above educationally and 303 (93.80%) were married. (Table 1).

**Table 1:** Sociodemographic characteristics of pregnant women. Addis Ababa, Ethiopia 2021.

Characteristics	Frequency	Percentage (%)
**Age**		
18–25	127	39.30
26–32	152	47.10
≥33	44	13.60
**Marital status**		
Other	20	6.20
Married	303	93.80
**Educational level**		
No formal education	26	8.05
Primary	95	29.41
Secondary and above	202	62.54
**Occupational status**		
Employed	91	28.20
Self–employed	93	28.80
Housewife	134	41.50
Student	5	1.50
**Perceived social support**		
Low	9	2.79
Moderate	110	34.06
High	204	63.15

### Obstetric and clinical characteristics

Among the majority of pregnant women who participated in the study were multigravida (221, 68.40%) and multiparous women (183, 6.70%). Only 62 (19.20%) reported their pregnancy was unplanned. The majority were captured in the study in the 2^nd^ (121, 37.50%) and 3^rd^ trimesters (140, 43.30%). A history of chronic illness was reported by 35 (10.80%) participants. A family history of mental illness was reported by 17 (5.30%) of the study women. Only two women in the study reported a history of mental illness. (Table 2).

**Table 2:** Obstetric and clinical characteristics of pregnant women. Addis Ababa, Ethiopia 2021.

Obstetric characteristics	Frequency	Percentage (%)
Primigravida	102	31.60
Multigravida	221	68.40
**Parity**		
Nulliparous	140	43.30
Multiparous	183	56.70
**Current pregnancy planned**		
No	62	19.20
Yes	261	80.80
**Current pregnancy wanted**		
No	20	6.20
Yes	303	93.80
**Gestational age**		
1^st^ trimester	62	19.20
2^nd^ trimester	121	37.50
3^rd^ trimester	140	43.30
History of chronic illness Yes	35	10.80
No	288	89.20
**Family history of mental illness**		
Yes	17	5.30
No	306	94.70
**Self-reported history of mental illness**		
Yes	2	0.62
No	321	99.38
**Perceived Health Status**		
Very good	97	30.00
Good	184	57.00
Moderate/ Bad/very bad	42	13.00

### Prevalence of anxiety among pregnant women and associated factors

**Fig 1:**
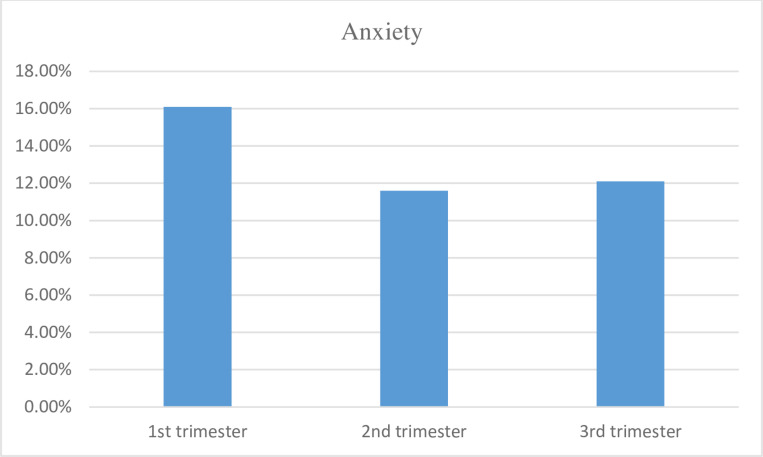
Prevalence of anxiety among pregnant women based on their gestational age, Addis Ababa, Ethiopia, 2021.

In this study, of the total of 323 study participants, 41 (12.70%, CI 9.30%–16.70%) had anxiety, and 282 (87.30%, CI 83.30%–90.70%) were not diagnosed to have anxiety. Participants in their 1^st^ trimester had a higher prevalence of anxiety 10 (16.10%), followed by the 3^rd^ trimester with a prevalence of 12.10% and 2^nd^ trimester at 11.60% (Fig 1).

The prevalence of anxiety among pregnant women was higher among ≥ 33 age groups (18.20%), followed by the 18–25 age group (14.20%); those with primary educational level (23.20%) were more affected; others (unmarried, divorced, separated and widowed) (55.00%) are more affected than the married; those with high (6.86%) and moderate (20.00%) social support are less affected compared to those with low social support (55.56%); those with unplanned pregnancy (32.26%) are more affected.

Factors significantly associated with anxiety in the unadjusted model include marital status, social support, and unplanned pregnancy. In the adjusted model, marital status, where single pregnant women were found to have a higher risk of anxiety than married ones, APR 2.29 (1.14–4.59), and those with an unplanned pregnancy had a higher risk of anxiety than those with a planned pregnancy APR 1.99 (1.04–3.81). Still, social support was not found to be significantly associated with anxiety in the final model. The unadjusted and adjusted prevalence ratio and Poisson regression output for associated factors are presented in the following table (Table 3).

**Table 3:** Factors associated with anxiety among pregnant women. Addis Ababa, Ethiopia, 2021.

	Anxiety		PR	APR
	Yes	No		
**Age**				
18–25	18	109	1	1
26–32	15	137	0.70 (0.37–1.33)	1.08 (0.50–2.37)
≥ 33	8	36	1.28 (0.60–2.74)	1.38 (0.64–2.94)
**Marital status**				
Single^a^	11	9	5.56 (3.29–9.37)	**2.29 (1.14**–**4.59)**
Married	30	273	1	**1**
**Educational status**				
No formal education	3	23	1	1
Primary	22	73	2.01 (0.65–6.20)	1.96 (0.63–6.12)
Secondary and above	16	186	0.69 (0.21–2.20)	1.08 (0.32–3.59)
**Employment**				
Unemployed	21	118	1.39 (0.78–2.46)	1.22 (0.64–2.32)
Employed	20	164	1	1
**Perceived social support**				
Low	5	4	8.10 (3.73–7.55)	2.39 (0.84–6.78)
Moderate	22	88	2.91 (1.55–5.47)	1.94 (0.95–3.93)
High	14	190	1	1
**Gestational Age**				
First Trimester	10	52	1.33 (0.64–2.74)	1.61 (0.81–3.16)
Second Trimester	14	107	0.95 (0.49–1.85)	1.20 (0.64–2.22)
Third Trimester	17	123	1	1
**Parity**				
Nulliparous	21	119	1.37 (0.77–2.43)	1.32 (0.65–2.70)
Multiparous	20	163	1	1
**Current pregnancy planned**				
No	20	42	4.01 (2.32–6.93)	**1.99 (1.04**–**3.81)**
Yes	21	240	1	**1**
**Family history of mental illness**				
Yes	3	14	1.42 (0.49–4.15)	1.48 (0.44–5.01)
No	38	268	1	1

^a^Single –never married, divorced, widowed, separated.

## Discussion

The prevalence of anxiety among pregnant women in this study was 12.70%; The overall prevalence observed in this study was in line with the studies conducted in urban South Africa (15% in early pregnancy)) [37], Uganda 13% [38], A meta-analysis 15.2% [10], higher than the study conducted in Arbaminch Ethiopia (4.20%) [13].The prevalence was lower than the findings of studies conducted in women visiting perinatal care in Dilla town, Ethiopia (32.2%) [25], Nigeria (37.5%) [39], and China (17%) [40].

This difference with our study could be due to the different measurement scales used in the studies, the socio-demographic difference, and the time the study was conducted in relation to the COVID-19 pandemic.

A significantly higher prevalence of anxiety was observed among women who had unplanned pregnancies and single-marital status.

Women with unplanned pregnancies were more likely to experience anxiety than women with planned pregnancies, which was also reported in previous studies [41–44]. Unplanned pregnancies put women and couples under unexpected pressure, with less readiness for parenthood. The stress may lead to mental health issues [39,45–48]. Stress may worsen if unplanned pregnancy is coupled with other life stressors, for example, the uncertainty of pregnancy outcome due to the COVID-19 pandemic [49].

In this study, those who reported being single had a higher prevalence of anxiety than married women. Similar to the results of this study, previous studies have shown that married women had a lower risk of anxiety disorder during pregnancy [3,50–52]. This demonstrates the crucial role that partners play in reducing or exacerbating anxiety during pregnancy and their important role in mediating the impact of unfavorable feelings and stressors related to pregnancy and the challenges associated with the adjustment to parenthood, as well as helping the mother prepare for childbirth [3,13].

The study used a structured questionnaire and standard anxiety measurement scale, which assessed the experience of pregnant women within the past week. Data collectors were trained, and the data collection was supervised regularly. Our limitations were that the anxiety measurement scale was previously validated in many LMICs, but the Amharic version of the tool used for assessing anxiety is not yet validated in Ethiopia. We were also underpowered to show a significant association with some variables due to resource constraints. We also anticipated a higher prevalence of anxiety because of the pandemic and wanted to do a speedy assessment.

## Conclusion

More than 1 in 10 women had anxiety during pregnancy. The prevalence of anxiety was about two-fold higher among single mothers and among mothers who had an unplanned pregnancy. The study suggests the importance of integrated mental health assessment and services in regular antenatal care, with an emphasis on identifying and caring for women at a disadvantage due to social and obstetric factors.
